# Supplementary material for: Galectin-3 promotes fibrosis in ovarian endometriosis
Source: PeerJ. 2024 Feb 14;12:e16922. doi: 10.7717/peerj.16922 (PMC10874174; doi:10.7717/peerj.16922)

Fig.3-α-SMA
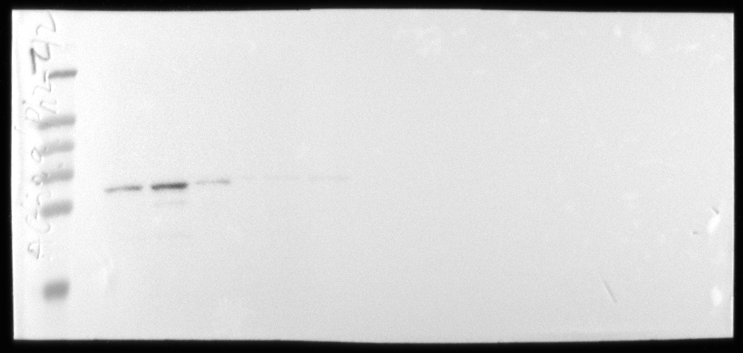


Fg.3-COL-1
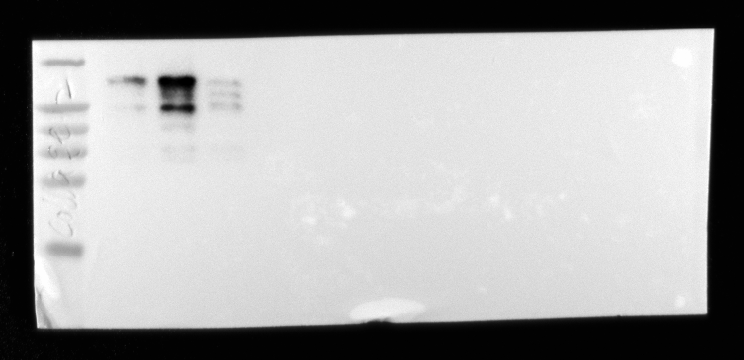


Fig.3-CTGF
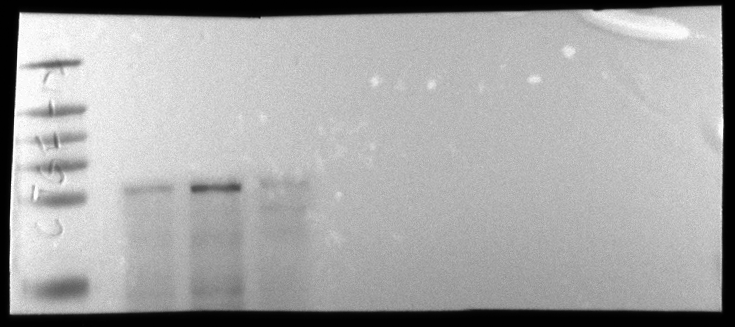


Fig.3-Gal-3
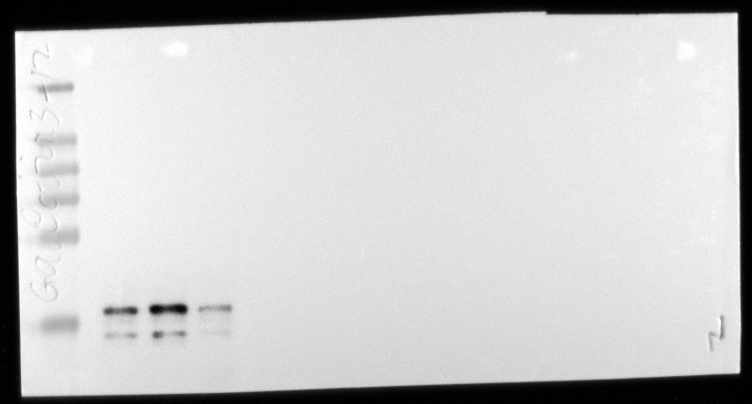


Fig.3-Actin
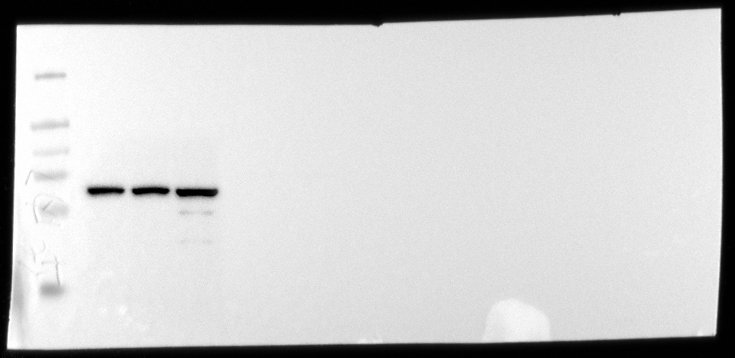


Fig.7-α-SMA
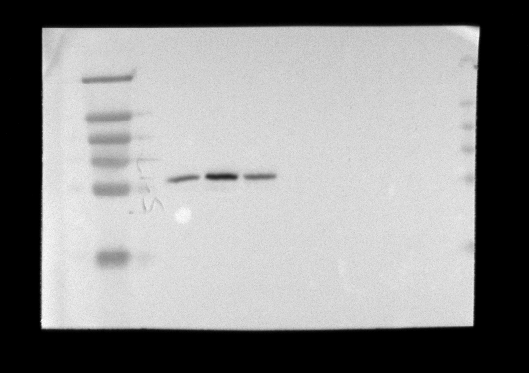


Fig.7-Actin
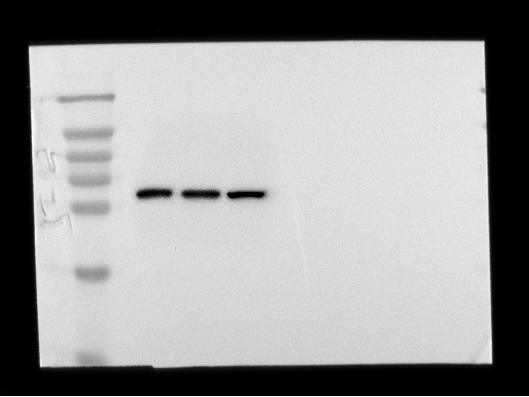


Fig.7-CTGF
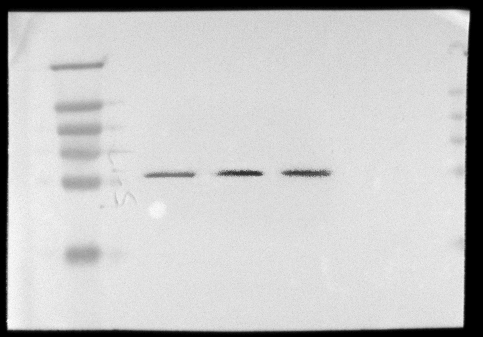


Fig.7-COL-1
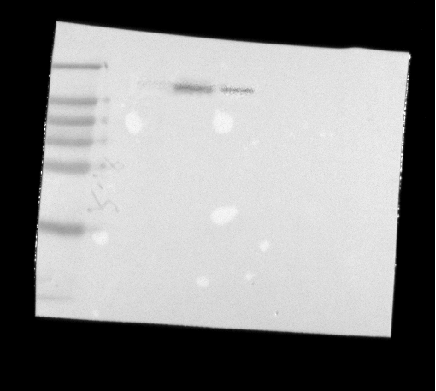


Fig.7-Gal-3
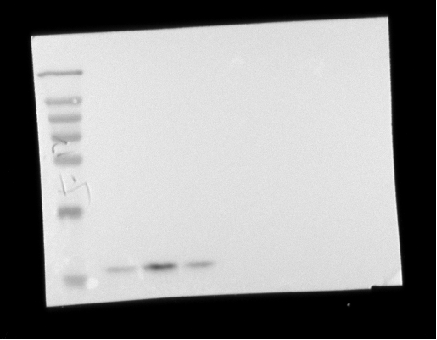


Fig.9-Actin
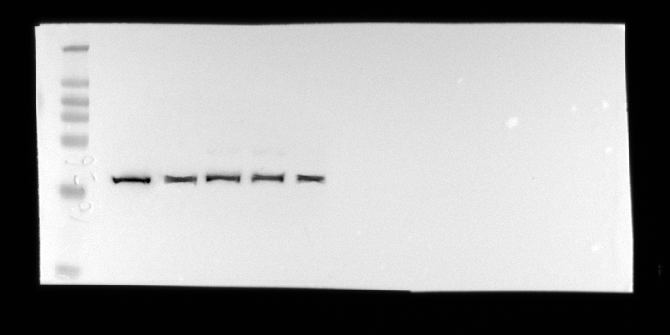


Fig.9-COL-1
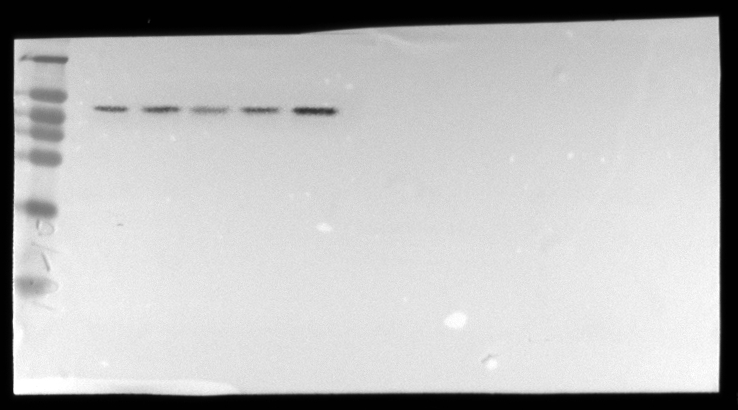


Fig.9-CTGF
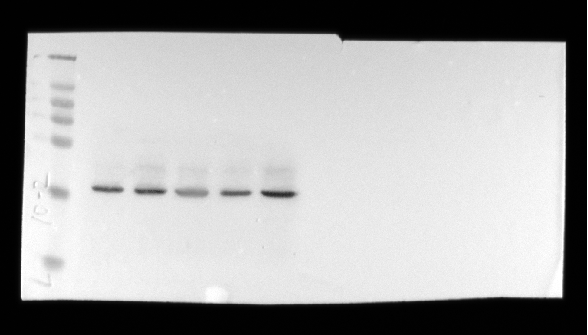


Fig.9-Gal-3
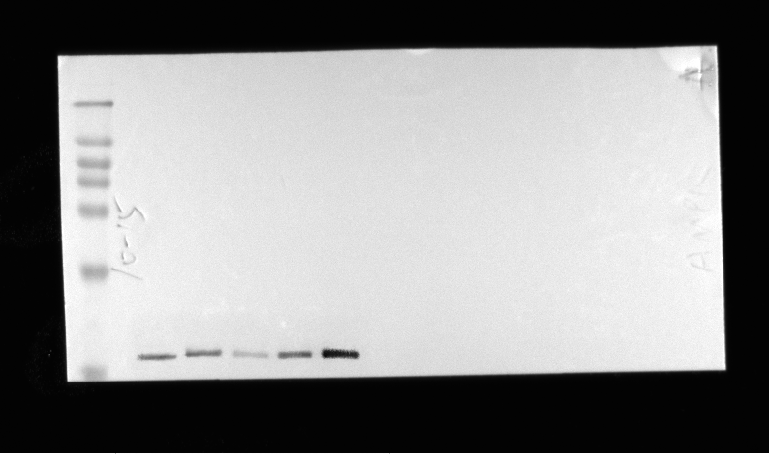


Fig.9-α-SMA
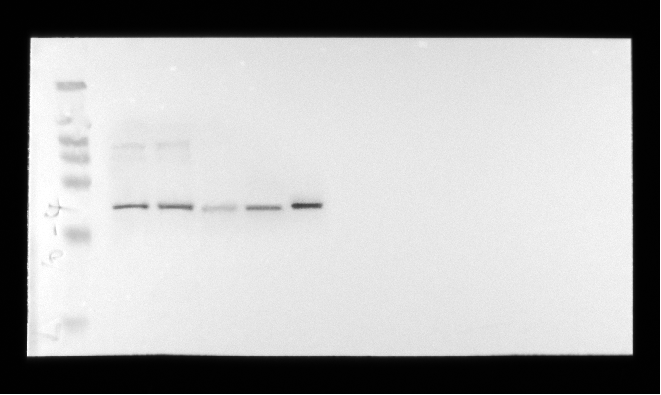

Supplement: Supplemental Information 2 [file peerj-12-16922-s002.doc]
